# Supplementary material for: Prevalence and Factors Associated with Common Mental Disorders and Posttraumatic Stress Disorder Among Healthcare Workers in a Reference Center for Infectious Diseases During the COVID-19 Pandemic: A Survey-Based Cross-Sectional Study
Source: Int J Environ Res Public Health. 2025 Feb 13;22(2):271. doi: 10.3390/ijerph22020271 (PMC11855729; doi:10.3390/ijerph22020271)

**Appendix. Supplementary data**

**Title:** Prevalence and factors associated with common mental disorders and posttraumatic stress disorder among healthcare workers in a reference center for infectious diseases during the COVID-19 pandemic: A survey-based cross-sectional study

**Table S1:** Univariate logistic regression analysis of factors associated with the GHQ-12 score among healthcare workers (2020 survey)

| **Characteristics** | | **2020 Survey** | | | | | | |
| --- | --- | --- | --- | --- | --- | --- | --- | --- |
|  |  | **GHQ-12 ≥ 5** | | **GHQ-12 <5** | |  |  |  |
|  |  | **n** | **%** | **n** | **%** | **Crude OR** | **95% CI** | **p value** |
| Sex |  |  |  |  |  |  |  |  |
|  | Female | 96 | 75.6 | 160 | 65.8 | 1.61 | 0.99-2.61 | 0.055 |
|  | Male | 31 | 24.4 | 83 | 34.2 | 1 | - |  |
| Age (per year increase) |  |  |  |  |  |  |  |  |
|  | Median (IQR) | 38 (30-45) | | 39 (32-46) | | 0.99 | 0.97-1.01 | 0.263 |
| Highest educational attainment | |  |  |  |  |  |  |  |
|  | Up to high school | 8 | 6.3 | 48 | 19.8 | 3.66 | 1.67-8.01 | 0.001 |
|  | University or higher | 119 | 93.7 | 195 | 80.2 | 1 | - |  |
| Marital Status | |  |  |  |  |  |  |  |
|  | Single, Separated, divorced or widowed | 75 | 59.1 | 123 | 50.8 | 1.40 | 0.90-2.15 | 0.133 |
|  | Married/living with a partner | 52 | 40.9 | 119 | 49.2 | 1 | - |  |
| Household monthly income | |  |  |  |  |  |  |  |
|  | Up to 2 minimum wage | 9 | 7.9 | 23 | 10.4 | 0.60 | 0-26-1.40 | 0.237 |
|  | 3-7 minimum wage | 51 | 44.7 | 115 | 52.0 | 0.68 | 0.42-1.10 | 0.114 |
|  | > 8 minimum wage | 54 | 47.4 | 83 | 37.6 | 1 | - |  |
| Religion |  |  |  |  |  |  |  |  |
|  | No | 23 | 18.1 | 42 | 17.3 | 1.06 | 0.60-1.85 | 0.843 |
|  | Yes | 104 | 81.9 | 201 | 82.7 | 1 | - |  |
| Living Alone | |  |  |  |  |  |  |  |
|  | No | 107 | 84.3 | 198 | 81.5 | 1 | - |  |
|  | Yes | 20 | 15.7 | 45 | 18.5 | 0.82 | 0.46-1.46 | 0.507 |
| Having Children (≤ 16 years) | |  |  |  |  |  |  |  |
|  | No | 84 | 66.1 | 140 | 57.6 | 1 | - |  |
|  | Yes | 43 | 33.9 | 103 | 42.4 | 0.70 | 0.44-1.09 | 0.112 |
| Social Network | |  |  |  |  |  |  |  |
|  | No | 6 | 4.7 | 15 | 6.2 | 0.75 | 0.29-1.99 | 0.569 |
|  | Yes | 121 | 95.3 | 228 | 93.8 | 1 | - |  |
| Self-reported history of chronic diseases | |  |  |  |  |  |  |  |
|  | No | 51 | 40.2 | 133 | 54.7 | 1 | - |  |
|  | Yes | 76 | 59.8 | 110 | 45.3 | 1.8 | 1.17-2.79 | 0.008 |
| Current smoking | |  |  |  |  |  |  |  |
|  | No | 120 | 95.2 | 221 | 93.2 | 1 | - |  |
|  | Yes | 6 | 4.8 | 16 | 6.8 | 0.69 | 0.26-1.81 | 0.452 |
| Having hobby | |  |  |  |  |  |  |  |
|  | No | 40 | 31.5 | 64 | 26.3 | 1.29 | 0.80-2.06 | 0.295 |
|  | Yes | 87 | 68.5 | 179 | 73.7 | 1 | - |  |
| Having pet | |  |  |  |  |  |  |  |
|  | No | 53 | 42.1 | 107 | 44.2 | 0.92 | 0.59-1.42 | 0.693 |
|  | Yes | 73 | 57.9 | 135 | 55.8 | 1 | - |  |
| Having regular physical activity | |  |  |  |  |  |  |  |
|  | No | 90 | 71.4 | 124 | 52.1 | 2.3 | 1.45-3.65 | <0.001 |
|  | Yes | 36 | 28.6 | 114 | 47.9 | 1 | - |  |
| Admitted to hospital in the 14 days before completing the survey | |  |  |  |  |  |  |  |
|  | No | 123 | 96.9 | 239 | 98.4 | 1 | - |  |
|  | Yes | 4 | 3.1 | 4 | 1.6 | 1.94 | 0.48-7.90 | 0.353 |
| COVID-19 related symptoms in the 14 days before completing the survey | |  |  |  |  |  |  |  |
|  | No | 54 | 42.5 | 143 | 58.8 | 1 | - |  |
|  | Yes | 73 | 57.5 | 100 | 41.2 | 1.93 | 1.25-2.99 | 0.003 |
| Has been performed a SARS-CoV-2  diagnostic test in the 14 days before completing the survey | | | | | |  |  |  |
|  | No | 93 | 73.2 | 169 | 69.5 | 1 | - |  |
|  | Yes | 34 | 26.8 | 74 | 30.5 | 0.84 | 0.52-1.35 | 0.460 |
| Formally diagnosed with COVID-19  by a physician | | | |  |  |  |  |  |
|  | No | 97 | 77.0 | 185 | 77.4 | 1 | - |  |
|  | Yes | 29 | 23.0 | 54 | 22.6 | 1.02 | 0.61-1.71 | 0.927 |
| Living with a high-risk person of getting  seriously ill from COVID-19 | | | | | |  |  |  |
|  | No | 71 | 56.4 | 161 | 66.5 | 1 | - |  |
|  | Yes | 55 | 43.6 | 81 | 33.5 | 1.54 | 0.99-2.40 | 0.056 |
| Has or have had any family members  with COVID-19 | | | |  |  |  |  |  |
|  | No | 78 | 61.4 | 161 | 66.8 | 1 | - |  |
|  | Yes | 49 | 38.6 | 80 | 33.2 | 1.26 | 0.81-1.98 | 0.304 |
| Have lost a family member or friend  with COVID-19 | | | |  |  |  |  |  |
|  | No | 73 | 57.9 | 160 | 65.8 | 1 | - |  |
|  | Yes | 53 | 42.1 | 83 | 34.2 | 1.4 | 0.90-2.18 | 0.136 |
| Weekly working hours | |  |  |  |  |  |  |  |
|  | < 40 hours | 48 | 37.8 | 136 | 56.0 | 1 | - |  |
|  | ≥ 40 hours | 79 | 62.2 | 107 | 44.0 | 2.09 | 1.35-3.25 | 0.001 |
| Development of work activities in the 14 days before completing the survey | |  |  |  |  |  |  |  |
|  | Exclusively remote | 21 | 16.6 | 17 | 7.0 | 1 | - |  |
|  | Hybrid | 37 | 29.1 | 36 | 14.8 | 0.83 | 0.38-1.83 | 0.647 |
|  | Entirely in person | 69 | 54.3 | 190 | 78.2 | 0.29 | 0.15-0.59 | 0.001 |
| Frontline health workers | |  |  |  |  |  |  |  |
|  | No | 76 | 59.8 | 103 | 42.4 | 1 | - |  |
|  | Yes | 51 | 40.2 | 140 | 57.6 | 0.49 | 0.32-0.76 | 0.002 |

2020 survey: One participant did not complete the GHQ-12; GHQ-12, the 12-item General Health Questionnaire; CI, confidence interval; OR, odds ratio; IQR, interquartile range. The variables "Development of work activities in the 14 days before completing the survey" and "Weekly working hours" were not included in the multivariate model due to collinearity with "frontline health workers".

**Table S2:** Univariate logistic regression analysis of factors associated with the GHQ-12 score among healthcare workers (2021 survey)

| **Characteristics** | | | **2021 Survey** | | | | | | | | |
| --- | --- | --- | --- | --- | --- | --- | --- | --- | --- | --- | --- |
|  |  |  | **GHQ-12 ≥5** | | | **GHQ-12 <5** | |  |  |  |  |
|  |  |  | **n** | **%** | | **n** | **%** | **Crude OR** | **95% CI** | **p value** |  |
| Sex |  | |  |  | |  |  |  |  |  |  |
|  | Female | | 41 | 80.4 | | 82 | 70.7 | 1.7 | 0.77-3.78 | 0.193 |  |
|  | Male | | 10 | 19.6 | | 34 | 29.3 | 1 | - |  |  |
| Age (per year increase) |  | |  |  | |  |  |  |  |  |  |
|  | Median (IQR) | | 42 (33-49.3) | | | 41 (34-49) | | 1.00 | 0.97-1.04 | 0.865 |  |
| Highest educational attainment | | |  |  | |  |  |  |  |  |  |
|  | Up to high school | | 4 | 7.8 | | 18 | 15.5 | 2.16 | 0.69-6.73 | 0.185 |  |
|  | University or higher | | 47 | 92.2 | | 98 | 84.5 | 1 | - |  |  |
| Marital Status | | |  |  | |  |  |  |  |  |  |
|  | Single, Separated, divorced or widowed | | 30 | 58.8 | | 45 | 38.8 | 2.25 | 1.15-4.41 | 0.018 |  |
|  | Married/living with a partner | | 21 | 41.2 | | 71 | 61.2 | 1 | - |  |  |
| Household monthly income | | |  |  | |  |  |  |  |  |  |
|  | Up to 2 minimum wage | | 4 | 8.3 | | 5 | 4.7 | 1.57 | 0.39-6.32 | 0.524 |  |
|  | 3-7 minimum wage | | 16 | 33.3 | | 47 | 43.9 | 0.67 | 0.32-1.38 | 0.278 |  |
|  | > 8 minimum wage | | 28 | 58.3 | | 55 | 51.4 | 1 | - |  |  |
| Religion |  | |  |  | |  |  |  |  |  |  |
|  | No | | 9 | 17.6 | | 14 | 12.1 | 1.56 | 0.63-3.88 | 0.338 |  |
|  | Yes | | 42 | 82.4 | | 102 | 87.9 | 1 | - |  |  |
| Living Alone | | |  |  | |  |  |  |  |  |  |
|  | No | | 44 | 86.3 | | 100 | 86.2 | 1 | - |  |  |
|  | Yes | | 7 | 13.7 | | 16 | 13.8 | 0.99 | 0.38-2.59 | 0.991 |  |
| Having Children (≤ 16 years) | | |  |  | |  |  |  |  |  |  |
|  | No | | 35 | 68.6 | | 65 | 56.0 | 1 | - |  |  |
|  | Yes | | 16 | 31.4 | | 51 | 44.0 | 0.58 | 0.29-1.17 | 0.128 |  |
| Social Network | | |  |  | |  |  |  |  |  |  |
|  | No | | 2 | 3.9 | | 7 | 6.0 | 0.64 | 0.13-3.17 | 0.580 |  |
|  | Yes | | 49 | 96.1 | | 109 | 94.0 | 1 | - |  |  |
| Self-reported history of chronic diseases | | |  |  | |  |  |  |  |  |  |
|  | No | | 14 | 28.0 | | 57 | 49.1 | 1 | - |  |  |
|  | Yes | | 36 | 72.0 | | 59 | 50.9 | 2.48 | 1.21-5.09 | 0.013 |  |
| Current smoking | | |  |  | |  |  |  |  |  |  |
|  | No | | 50 | 98.0 | | 109 | 94.0 | 1 | - |  |  |
|  | Yes | | 1 | 2.0 | | 7 | 6.0 | 0.31 | 0.04-2.60 | 0.281 |  |
| Having hobby | | |  |  | |  |  |  |  |  |  |
|  | No | | 20 | 39.2 | | 35 | 30.4 | 1.48 | 0.74-2.94 | 0.269 |  |
|  | Yes | | 31 | 60.8 | | 80 | 69.6 | 1 | - |  |  |
| Having pet | | |  |  | |  |  |  |  |  |  |
|  | No | | 20 | 39.2 | | 48 | 41.4 | 0.91 | 0.47-1.79 | 0.793 |  |
|  | Yes | | 31 | 60.8 | | 68 | 58.6 | 1 | - |  |  |
| Having regular physical activity | | |  |  | |  |  |  |  |  |  |
|  | No | | 26 | 52.0 | | 62 | 53.4 | 0.94 | 0.49-1.83 | 0.864 |  |
|  | Yes | | 24 | 48.0 | | 54 | 46.6 | 1 | - |  |  |
| Admitted to hospital in the 14 days before completing the survey | | |  |  | |  |  |  |  |  |  |
|  | No | | 51 | 100.0 | | 114 | 98.3 | 1 | - |  |  |
|  | Yes | | 0 | - | | 2 | 1.7 | 0 | - | 0.999 |  |
| COVID-19 related symptoms in the 14 days before completing the survey | | |  |  | |  |  |  |  |  |  |
|  | No | | 17 | 33.3 | | 59 | 50.9 | 1 | - |  |  |
|  | Yes | | 34 | 66.7 | | 57 | 49.1 | 2.07 | 1.04-4.11 | 0.038 |  |
| Has been performed a SARS-CoV-2 diagnostic test in the 14 days before completing the survey | |  |  |  |  |  |  |  |  |  |  |
|  | No | | 41 | 80.4 | | 107 | 92.2 | 1 | - |  |  |
|  | Yes | | 10 | 19.6 | | 9 | 7.8 | 2.9 | 1.10-7.65 | 0.031 |  |
| Formally diagnosed with COVID-19 by a physician | | | | |  |  |  |  |  |  |  |
|  | No | | 29 | 56.9 | | 68 | 58.6 | 1 | - |  |  |
|  | Yes | | 22 | 43.1 | | 48 | 41.4 | 1.08 | 0.55-2.09 | 0.832 |  |
| Living with a high-risk person of getting seriously ill from COVID-19 | |  |  |  |  |  |  |  |  |  |  |
|  | No | | 25 | 49.0 | | 71 | 61.2 | 1 | - |  |  |
|  | Yes | | 26 | 51.0 | | 45 | 38.8 | 1.64 | 0.85-3.19 | 0.144 |  |
| Has or have had any family members with COVID-19 | | | | |  |  |  |  |  |  |  |
|  | No | | 12 | 23.5 | | 39 | 33.6 | 1 | - |  |  |
|  | Yes | | 39 | 76.5 | | 77 | 66.4 | 1.65 | 0.78-3.50 | 0.195 |  |
| Have lost a family member or friend with COVID-19 | | | | |  |  |  |  |  |  |  |
|  | No | | 16 | 31.4 | | 53 | 45.7 | 1 | - |  |  |
|  | Yes | | 35 | 68.6 | | 63 | 54.3 | 1.84 | 0.92-3.69 | 0.086 |  |
| Weekly working hours | | |  |  | |  |  |  |  |  |  |
|  | < 40 hours | | 23 | 45.1 | | 64 | 55.2 | 1 | - |  |  |
|  | ≥ 40 hours | | 28 | 54.9 | | 52 | 44.8 | 1.50 | 0.77-2.90 | 0.231 |  |
| Development of work activities in the 14 days before completing the survey | | |  |  | |  |  |  |  |  |  |
|  | Exclusively remote | | 4 | 7.8 | | 7 | 6.0 | 1 | - |  |  |
|  | Hybrid | | 14 | 27.5 | | 19 | 16.4 | 1.29 | 0.32-5.28 | 0.724 |  |
|  | Entirely in person | | 33 | 64.7 | | 90 | 77.6 | 0.64 | 0.18-2.34 | 0.501 |  |
| Frontline health workers | | |  |  | |  |  |  |  |  |  |
|  | No | | 28 | 54.9 | | 40 | 34.5 | 1 | - |  |  |
|  | Yes | | 23 | 45.1 | | 76 | 65.5 | 0.43 | 0.22-0.85 | 0.014 |  |

GHQ-12, the 12-item General Health Questionnaire; CI, confidence interval; OR, odds ratio; IQR, interquartile range

The variables "Development of work activities in the 14 days before completing the survey" and "Weekly working hours" were not included in the multivariate model due to collinearity with "frontline health workers".

**Table S3:** Univariate logistic regression analysis of factors associated with the IES-R score among healthcare workers (2020 survey)

| **Characteristics** | | **2020 Survey** | | | | | | | |  |
| --- | --- | --- | --- | --- | --- | --- | --- | --- | --- | --- |
|  |  | **IES-R ≥ 33** | | **IES-R < 33** | |  |  |  |  | |
|  |  | **n** | **%** | **n** | **%** | **Crude OR** | **95% CI** | **p value** |  | |
| Sex |  |  |  |  |  |  |  |  |  | |
|  | Female | 74 | 78.7 | 183 | 66.3 | 1.88 | 1.08-3.27 | 0.025 |  | |
|  | Male | 20 | 21.3 | 93 | 33.7 | 1 | - |  |  | |
| Age (per year increase) |  |  |  |  |  |  |  |  |  | |
|  | Median (IQR) | 37 (31.8-44) | | 40 (32-47) | | 0.97 | 0.95-0.99 | 0.024 |  | |
| Highest educational attainment | |  |  |  |  |  |  |  |  | |
|  | Up to high school | 11 | 11.7 | 45 | 16.3 | 1.47 | 0.73-2.98 | 0.284 |  | |
|  | University or higher | 83 | 88.3 | 231 | 83.7 | 1 | - |  |  | |
| Marital Status | |  |  |  |  |  |  |  |  | |
|  | Single, Separated,  divorced or widowed | 56 | 59.6 | 142 | 51.6 | 1.38 | 0.86-2.22 | 0.184 |  | |
|  | Married/living with a partner | 38 | 40.4 | 133 | 48.4 | 1 | - |  |  | |
|  |  |  |  |  |  |  |  |  |  | |
| Household monthly income | |  |  |  |  |  |  |  |  | |
|  | Up to 2 minimum wage | 6 | 7.2 | 26 | 10.4 | 0.69 | 0.26-1.82 | 0.457 |  | |
|  | 3-7 minimum wage | 43 | 51.8 | 123 | 49.0 | 1.05 | 0.62-1.77 | 0.858 |  | |
|  | > 8 minimum wage | 34 | 41.0 | 102 | 40.6 | 1 | - |  |  | |
| Religion |  |  |  |  |  |  |  |  |  | |
|  | No | 18 | 19.1 | 47 | 17.0 | 1.15 | 0.63-2.11 | 0.641 |  | |
|  | Yes | 76 | 80.9 | 229 | 83.0 | 1 | - |  |  | |
| Living Alone | |  |  |  |  |  |  |  |  | |
|  | No | 83 | 88.3 | 222 | 80.4 | 1 | - |  |  | |
|  | Yes | 11 | 11.7 | 54 | 19.6 | 0.55 | 0.27-1.09 | 0.087 |  | |
| Having Children (≤ 16 years) | |  |  |  |  |  |  |  |  | |
|  | No | 53 | 56.4 | 171 | 62.0 | 1 | - |  |  | |
|  | Yes | 41 | 43.6 | 105 | 38.0 | 1.26 | 0.78-2.02 | 0.340 |  | |
| Social Network | |  |  |  |  |  |  |  |  | |
|  | No | 4 | 4.3 | 17 | 6.2 | 0.68 | 0.22-2.07 | 0.493 |  | |
|  | Yes | 90 | 95.7 | 259 | 93.8 | 1 | - |  |  | |
| Self-reported history of chronic diseases | |  |  |  |  |  |  |  |  | |
|  | No | 36 | 38.3 | 148 | 53.6 | 1 | - |  |  | |
|  | Yes | 58 | 61.7 | 128 | 46.4 | 1.86 | 1.15-3.01 | 0.011 |  | |
| Current smoking | |  |  |  |  |  |  |  |  | |
|  | No | 89 | 94.7 | 252 | 93.7 | 1 | - |  |  | |
|  | Yes | 5 | 5.3 | 17 | 6.3 | 0.83 | 0.30-2.32 | 0.727 |  | |
| Having hobby | |  |  |  |  |  |  |  |  | |
|  | No | 25 | 26.9 | 79 | 28.6 | 0.92 | 0.54-1.55 | 0.747 |  | |
|  | Yes | 68 | 73.1 | 197 | 71.4 | 1 | - |  |  | |
| Having pet | |  |  |  |  |  |  |  |  | |
|  | No | 34 | 37.0 | 125 | 45.5 | 0.7 | 0.43-1.14 | 0.155 |  | |
|  | Yes | 58 | 63.0 | 150 | 54.5 | 1 | - |  |  | |
| Having regular physical activity | |  |  |  |  |  |  |  |  | |
|  | No | 60 | 65.2 | 154 | 56.8 | 1.43 | 0.87-2.33 | 0.158 |  | |
|  | Yes | 32 | 34.8 | 117 | 43.2 | 1 | - |  |  | |
| Admitted to hospital in the 14 days before completing the survey | |  |  |  |  |  |  |  |  | |
|  | No | 91 | 96.8 | 271 | 98.2 | 1 | - |  |  | |
|  | Yes | 3 | 3.2 | 5 | 1.8 | 1.79 | 0.42-7.62 | 0.433 |  | |
| COVID-19 related symptoms in the 14 days before completing the survey | |  |  |  |  |  |  |  |  | |
|  | No | 37 | 39.4 | 160 | 58.0 | 1 | - |  |  | |
|  | Yes | 57 | 60.6 | 116 | 42.0 | 2.13 | 1.32-3.43 | 0.002 |  | |
| Has been performed a SARS-CoV-2  diagnostic test in the 14 days before completing the survey | |  |  |  |  |  |  |  |  | |
|  | No | 71 | 75.5 | 190 | 68.8 | 1 | - |  |  | |
|  | Yes | 23 | 24.5 | 86 | 31.2 | 0.72 | 0.42-1.22 | 0.220 |  | |
| Formally diagnosed with COVID-19  by a physician | | |  |  |  |  |  |  |  | |
|  | No | 70 | 75.3 | 211 | 77.6 | 1 | - |  |  | |
|  | Yes | 23 | 24.7 | 61 | 22.4 | 1.14 | 0.66-1.97 | 0.649 |  | |
| Living with a high-risk person of  getting seriously ill from COVID-19 | | |  |  |  |  |  |  |  | |
|  | No | 48 | 52.2 | 183 | 66.5 | 1 | - |  |  | |
|  | Yes | 44 | 47.8 | 92 | 33.5 | 1.82 | 1.13-2.95 | 0.014 |  | |
| Has or have had any family members  with COVID-19 | |  |  |  |  |  |  |  |  | |
|  | No | 53 | 57.0 | 185 | 67.5 | 1 | - |  |  | |
|  | Yes | 40 | 43.0 | 89 | 32.5 | 1.57 | 0.97-2.54 | 0.067 |  | |
| Have lost a family member or friend  with COVID-19 | |  |  |  |  |  |  |  |  | |
|  | No | 49 | 53.3 | 183 | 66.3 | 1 | - |  |  | |
|  | Yes | 43 | 46.7 | 93 | 33.7 | 1.73 | 1.07-2.79 | 0.026 |  | |
| Weekly working hours | |  |  |  |  |  |  |  |  | |
|  | < 40 hours | 35 | 37.2 | 149 | 54.0 | 1 | - |  |  | |
|  | ≥ 40 hours | 59 | 62.8 | 127 | 46.0 | 1.98 | 1.22-3.20 | 0.005 |  | |
| Development of work activities in the 14 days before completing the survey | |  |  |  |  |  |  |  |  | |
|  | Exclusively remote | 14 | 14.9 | 24 | 8.7 | 1 | - |  |  | |
|  | Hybrid | 23 | 24.5 | 50 | 18.1 | 0.79 | 0.35-1.80 | 0.572 |  | |
|  | Entirely in person | 57 | 60.6 | 202 | 73.2 | 0.48 | 0.24-0.99 | 0.049 |  | |
| Frontline health workers | |  |  |  |  |  |  |  |  | |
|  | No | 50 | 53.2 | 128 | 46.4 | 1 | - |  |  | |
|  | Yes | 44 | 46.8 | 148 | 53.6 | 0.76 | 0.48-1.22 | 0.254 |  | |

2020 survey: One participant did not complete the IES-R form; IES-R, The Impact of Events Scale-Revised; CI, confidence interval; OR, odds ratio; IQR, interquartile range.

The variables "Development of work activities in the 14 days before completing the survey" and "Weekly working hours" were not included in the multivariate model due to collinearity with "frontline health workers".

**Table S4:** Univariate logistic regression analysis of factors associated with the IES-R score among healthcare workers (2021 survey)

| **Characteristics** | | **2021 Survey** | | | | | | | |
| --- | --- | --- | --- | --- | --- | --- | --- | --- | --- |
|  |  | **IES-R ≥ 33** | | **IES-R < 33** | |  |  |  |  |
|  |  | **n** | **%** | **n** | **%** | **Crude OR** | **95% CI** | **p value** |  |
| Sex |  |  |  |  |  |  |  |  |  |
|  | Female | 44 | 81.5 | 77 | 69.4 | 1.94 | 0.88-4.31 | 0.102 |  |
|  | Male | 10 | 18.5 | 34 | 30.6 | 1 | - |  |  |
| Age (per year increase) |  |  |  |  |  |  |  |  |  |
|  | Median (IQR) | 39 (31-47) | | 41.5 (36-49.3) | | 0.97 | 0.94-0.99 | 0.080 |  |
| Highest educational attainment | |  |  |  |  |  |  |  |  |
|  | Up to high school | 8 | 14.8 | 14 | 12.6 |  |  | 0.696 |  |
|  | University or higher | 46 | 85.2 | 97 | 87.4 | 1 | - |  |  |
| Marital Status | |  |  |  |  |  |  |  |  |
|  | Single, Separated,  divorced or widowed | 27 | 50.0 | 47 | 42.3 | 1.36 | 0.71-2.62 | 0.354 |  |
|  | Married/living with a partner | 27 | 50.0 | 64 | 57.7 | 1 | - |  |  |
|  |  |  |  |  |  |  |  |  |  |
| Household monthly income | |  |  |  |  |  |  |  |  |
|  | Up to 2 minimum wage | 5 | 9.8 | 4 | 3.9 | 2.8 | 0.69-11.32 | 0.149 |  |
|  | 3-7 minimum wage | 21 | 41.2 | 42 | 41.2 | 1.12 | 0.55-2.27 | 0.753 |  |
|  | > 8 minimum wage | 25 | 49.0 | 56 | 54.9 | 1 | - |  |  |
| Religion |  |  |  |  |  |  |  |  |  |
|  | No | 8 | 14.8 | 15 | 13.5 | 1.11 | 0.44-2.81 | 0.821 |  |
|  | Yes | 46 | 85.2 | 96 | 86.5 | 1 | - |  |  |
| Living Alone | |  |  |  |  |  |  |  |  |
|  | No | 47 | 87.0 | 95 | 85.6 | 1 | - |  |  |
|  | Yes | 7 | 13.0 | 16 | 14.4 | 0.88 | 0.34-2.30 | 0.801 |  |
| Having Children (≤ 16 years) | |  |  |  |  |  |  |  |  |
|  | No | 38 | 70.4 | 61 | 55.0 | 1 | - |  |  |
|  | Yes | 16 | 29.6 | 50 | 45.0 | 0.51 | 0.26-1.03 | 0.060 |  |
| Social Network | |  |  |  |  |  |  |  |  |
|  | No | 2 | 3.7 | 7 | 6.3 | 0.57 | 0.12-2.85 | 0.495 |  |
|  | Yes | 52 | 96.3 | 104 | 93.7 | 1 | - |  |  |
| Self-reported history of chronic diseases | |  |  |  |  |  |  |  |  |
|  | No | 20 | 37.0 | 51 | 46.4 | 1 | - |  |  |
|  | Yes | 34 | 63.0 | 59 | 53.6 | 1.47 | 0.75-2.86 | 0.258 |  |
| Current smoking | |  |  |  |  |  |  |  |  |
|  | No | 51 | 94.4 | 106 | 95.5 | 1 | - |  |  |
|  | Yes | 3 | 5.6 | 5 | 4.5 | 1.25 | 0.29-5.42 | 0.768 |  |
| Having hobby | |  |  |  |  |  |  |  |  |
|  | No | 21 | 38.9 | 33 | 30.0 | 1.49 | 0.75-2.94 | 0.256 |  |
|  | Yes | 33 | 61.1 | 77 | 70.0 | 1 | - |  |  |
| Having pet | |  |  |  |  |  |  |  |  |
|  | No | 19 | 35.2 | 49 | 44.1 | 0.69 | 0.35-1.35 | 0.274 |  |
|  | Yes | 35 | 64.8 | 62 | 55.9 | 1 | - |  |  |
| Having regular physical activity | |  |  |  |  |  |  |  |  |
|  | No | 20 | 37.0 | 66 | 60.0 | 0.39 | 0.20-0.77 | 0.006 |  |
|  | Yes | 34 | 63.0 | 44 | 40.0 | 1 | - |  |  |
| Admitted to hospital in the 14 days before completing the survey | |  |  |  |  |  |  |  |  |
|  | No | 53 | 98.1 | 110 | 99.1 | 1 | - |  |  |
|  | Yes | 1 | 1.9 | 1 | 0.9 | 2.08 | 0.13-33.83 | 0.608 |  |
| COVID-19 related symptoms in the 14 days before completing the survey | |  |  |  |  |  |  |  |  |
|  | No | 22 | 40.7 | 53 | 47.7 | 1 | - |  |  |
|  | Yes | 32 | 59.3 | 58 | 52.3 | 1.33 | 0.69-2.57 | 0.397 |  |
| Has been performed a SARS-CoV-2  diagnostic test in the 14 days before completing the survey | |  |  |  |  |  |  |  |  |
|  | No | 48 | 88.9 | 99 | 89.2 | 1 | - |  |  |
|  | Yes | 6 | 11.1 | 12 | 10.8 | 1.03 | 0.37-2.91 | 0.954 |  |
| Formally diagnosed with COVID-19 by a physician | | | | | | | | | |
|  | No | 34 | 63.0 | 61 | 55.0 | 1 | - |  |  |
|  | Yes | 20 | 37.0 | 50 | 45.0 | 0.72 | 0.37-1.40 | 0.330 |  |
| Living with a high-risk person of getting seriously ill from COVID-19 | | | | | | | | | |
|  | No | 27 | 50.0 | 68 | 61.3 | 1 | - |  |  |
|  | Yes | 27 | 50.0 | 43 | 38.7 | 1.58 | 0.82-3.05 | 0.171 |  |
| Has or have had any family members  with COVID-19 | |  |  |  |  |  |  |  |  |
|  | No | 13 | 24.1 | 36 | 32.4 | 1 | - |  |  |
|  | Yes | 41 | 75.9 | 75 | 67.6 | 1.51 | 0.72-3.17 | 0.272 |  |
| Have lost a family member or friend  with COVID-19 | |  |  |  |  |  |  |  |  |
|  | No | 21 | 38.9 | 48 | 43.2 | 1 | - |  |  |
|  | Yes | 33 | 61.1 | 63 | 56.8 | 1.2 | 0.62-2.33 | 0.595 |  |
| Weekly working hours | |  |  |  |  |  |  |  |  |
|  | < 40 hours | 32 | 61.5 | 54 | 50.0 | 1 | - |  |  |
|  | ≥ 40 hours | 20 | 38.5 | 54 | 50.0 | 0.63 | 0.32-1.23 | 0.172 |  |
| Development of work activities in the 14 days before completing the survey | |  |  |  |  |  |  |  |  |
|  | Exclusively remote | 1 | 1.9 | 10 | 9.0 | 1 | - |  |  |
|  | Hybrid | 10 | 18.5 | 22 | 19.8 | 4.55 | 0.51-40.51 | 0.175 |  |
|  | Entirely in person | 43 | 79.6 | 79 | 71.2 | 5.44 | 0.67-43.96 | 0.112 |  |
| Frontline health workers | |  |  |  |  |  |  |  |  |
|  | No | 21 | 38.9 | 46 | 41.4 | 1 | - |  |  |
|  | Yes | 33 | 61.1 | 65 | 58.6 | 1.11 | 0.57-2.16 | 0.754 |  |

2021 survey: Two participants did not complete the IES-R form. IES-R, The Impact of Events Scale-Revised; CI, confidence interval; OR, odds ratio; IQR, interquartile range.

The variables "Development of work activities in the 14 days before completing the survey" and "Weekly working hours" were not included in the multivariate model due to collinearity with "frontline health workers".


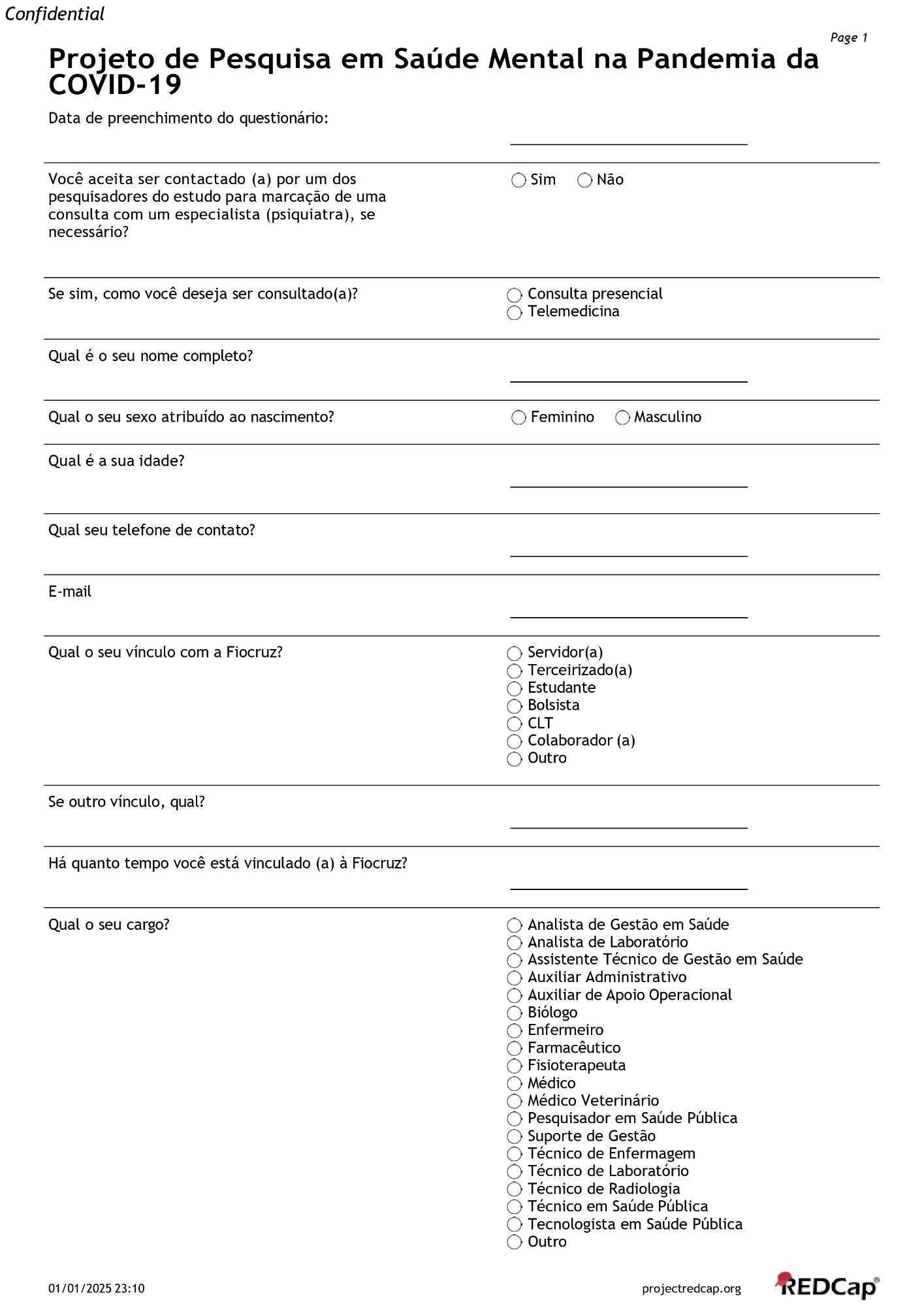


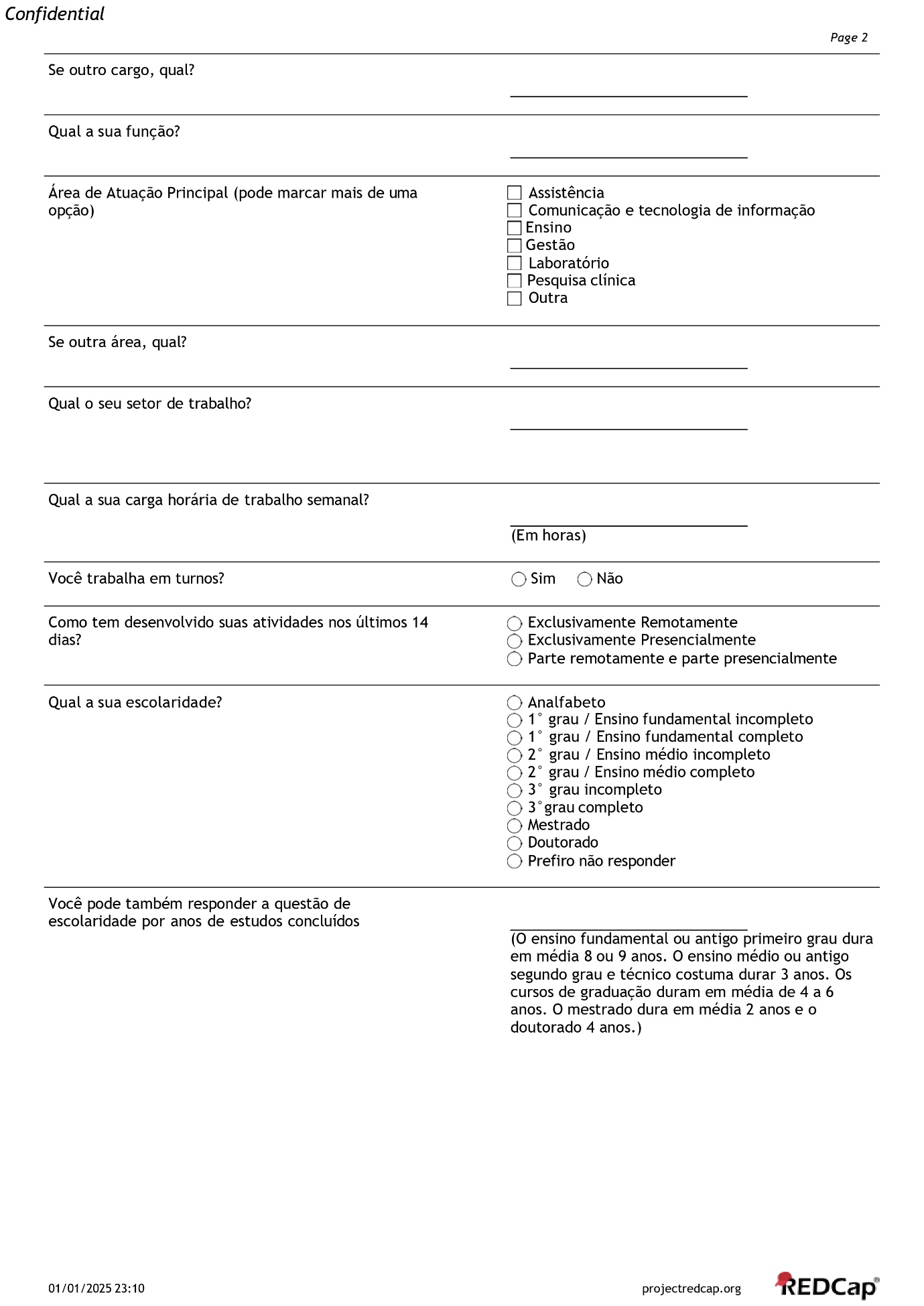

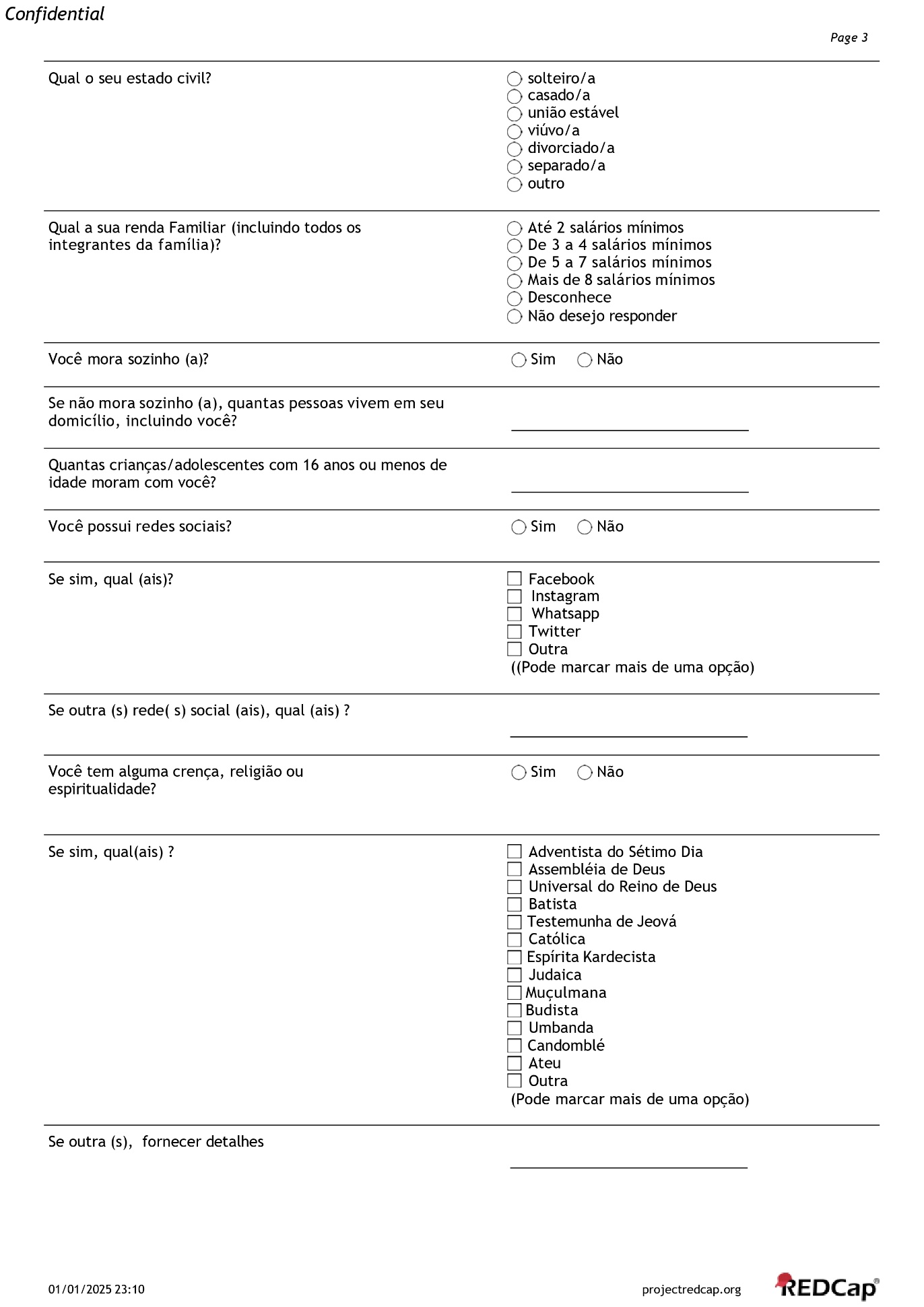

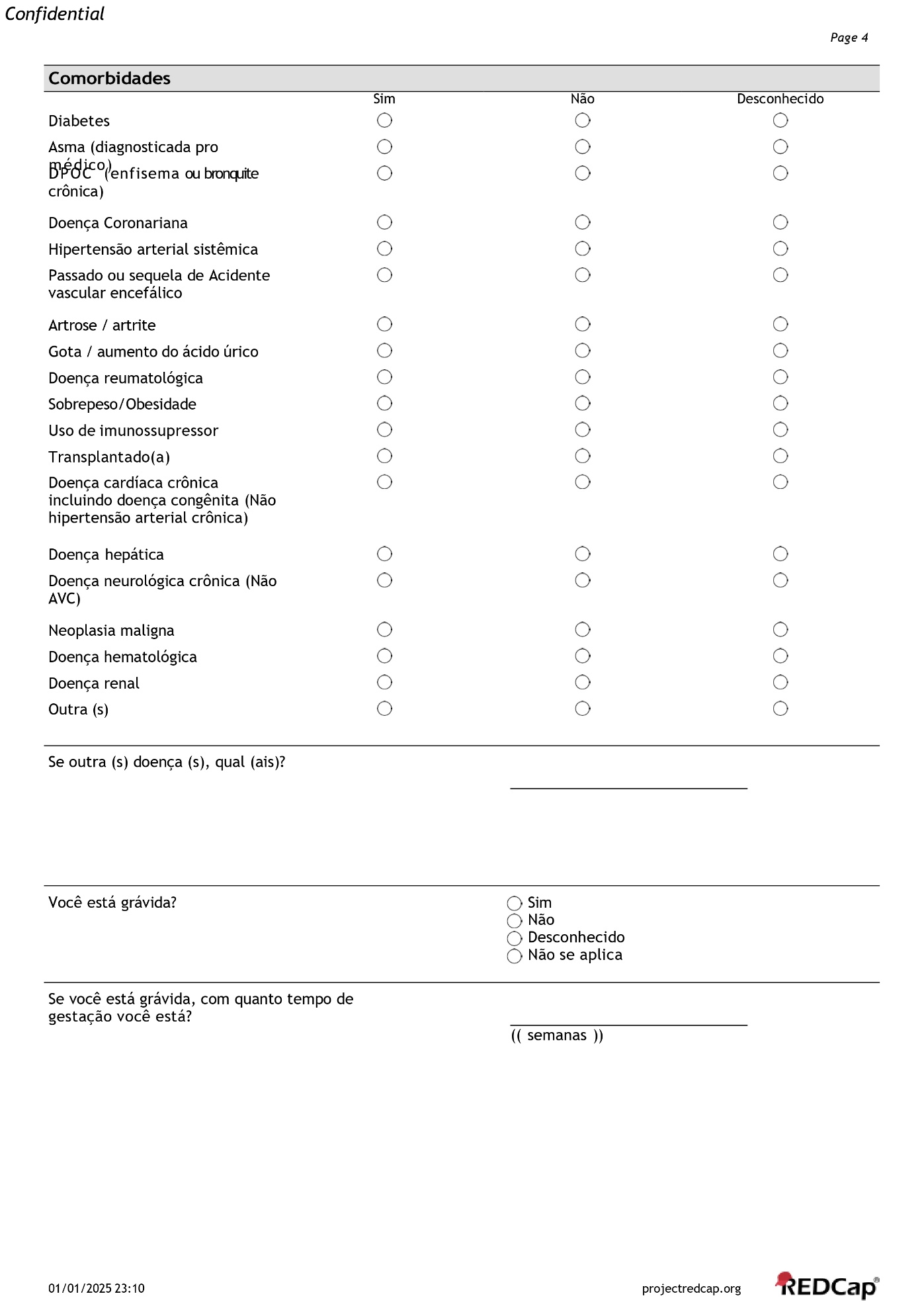

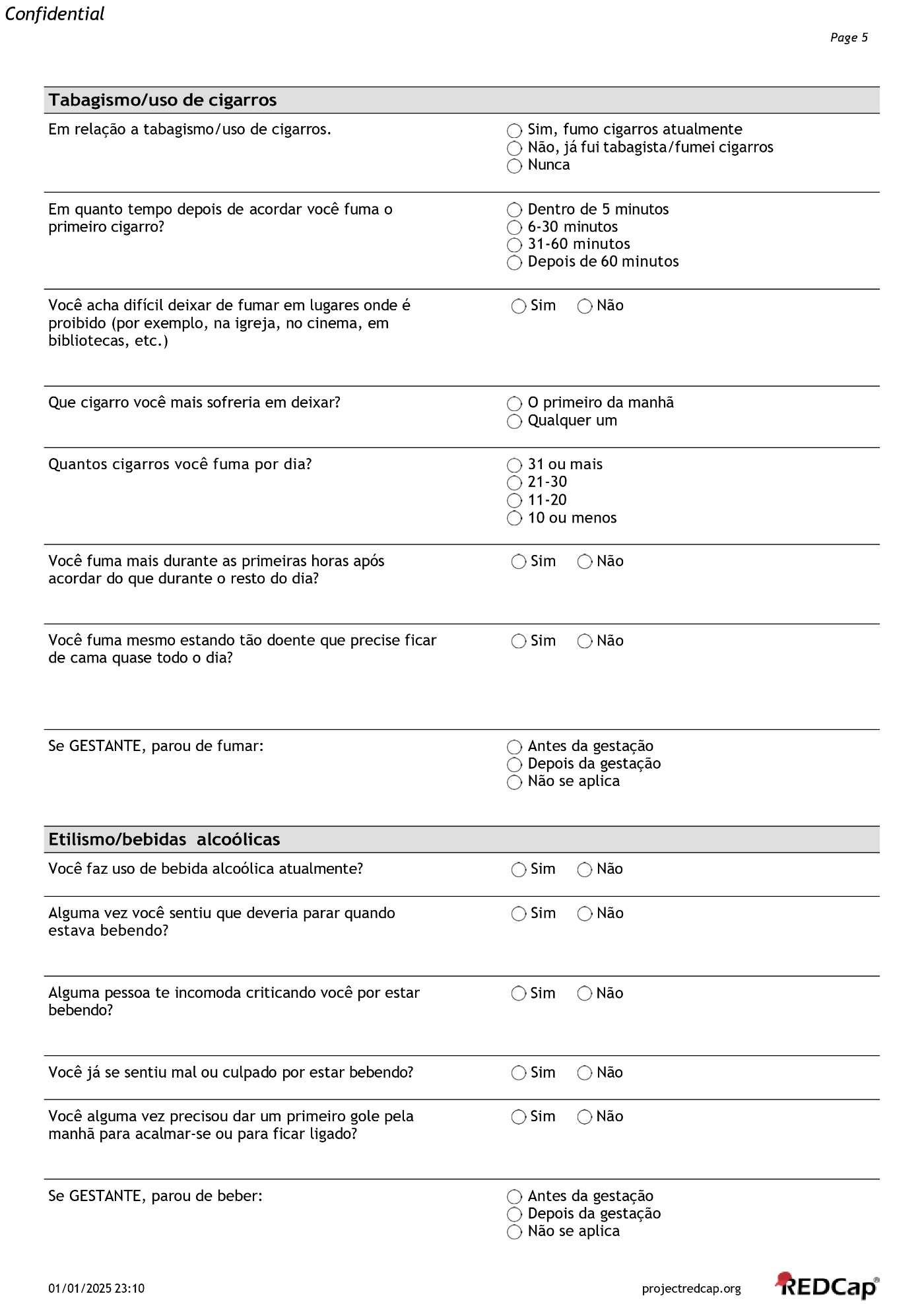

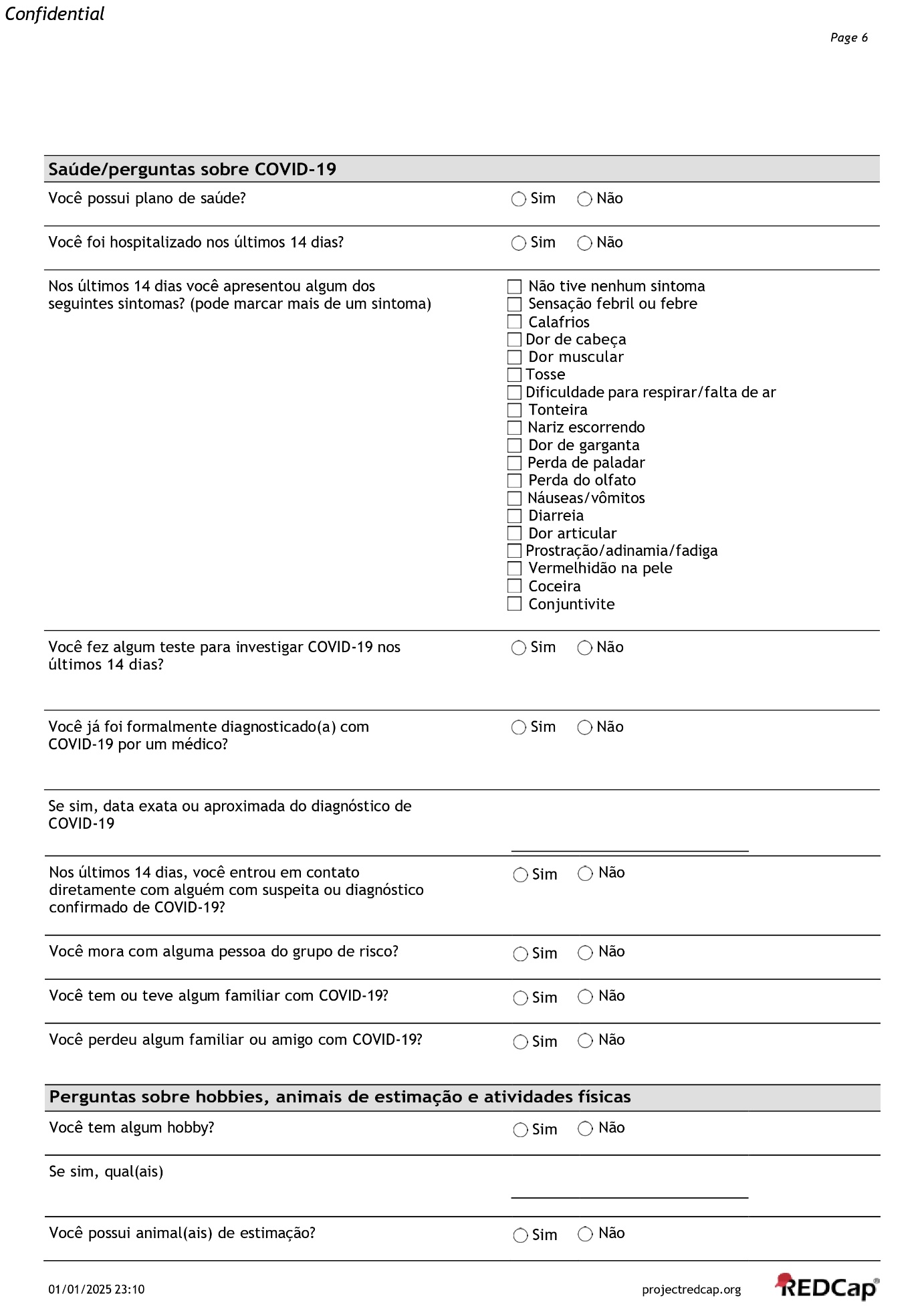

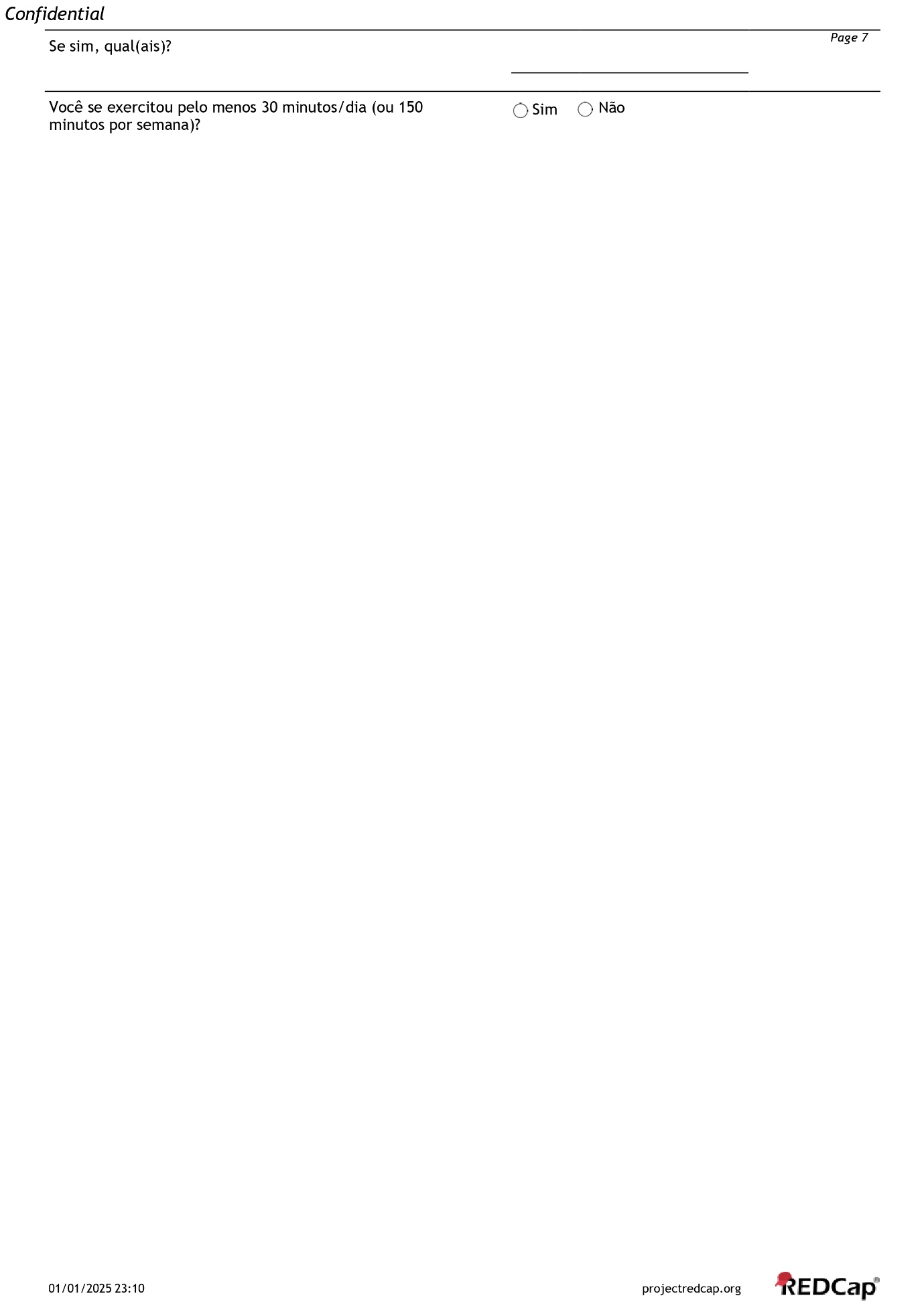

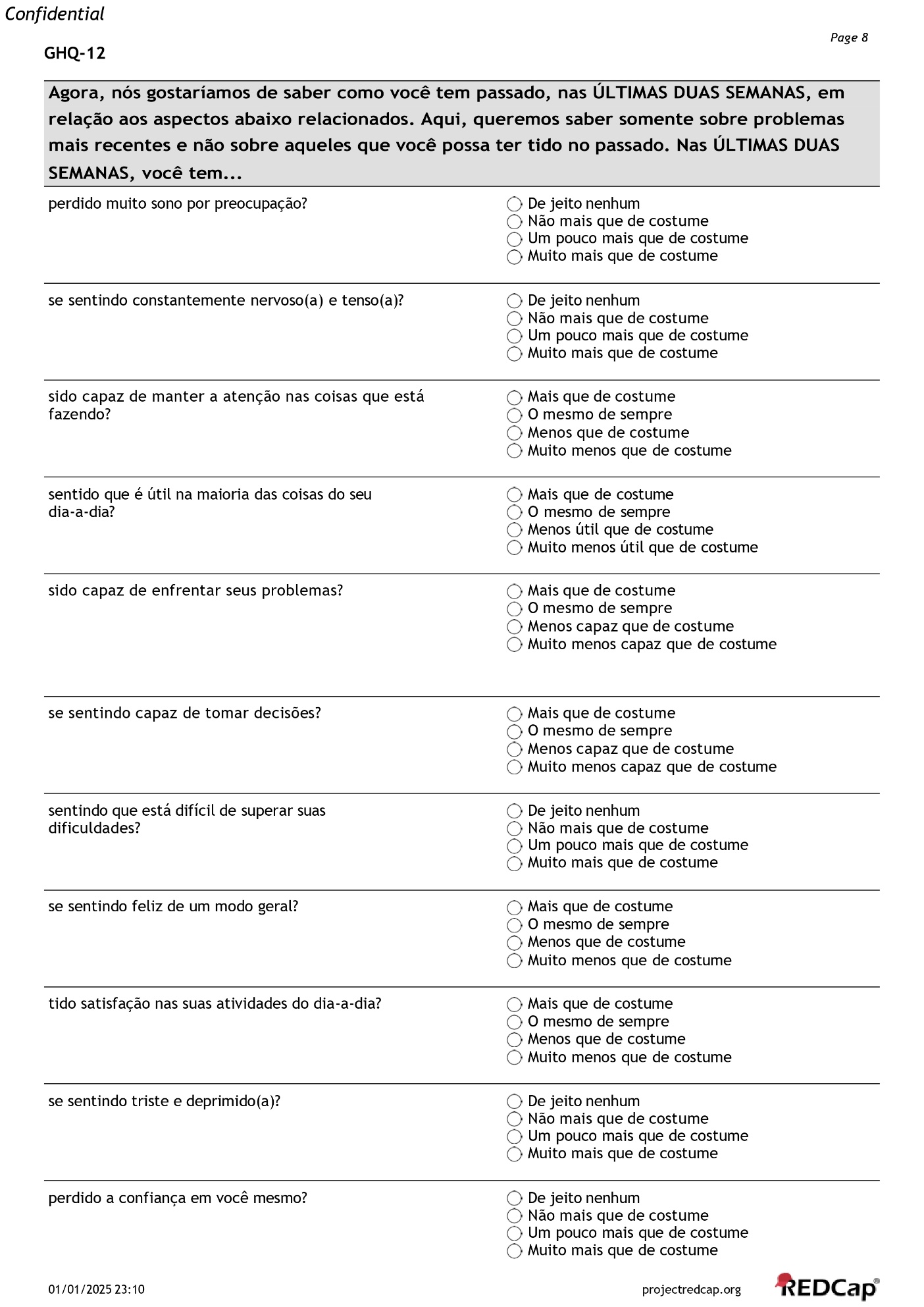

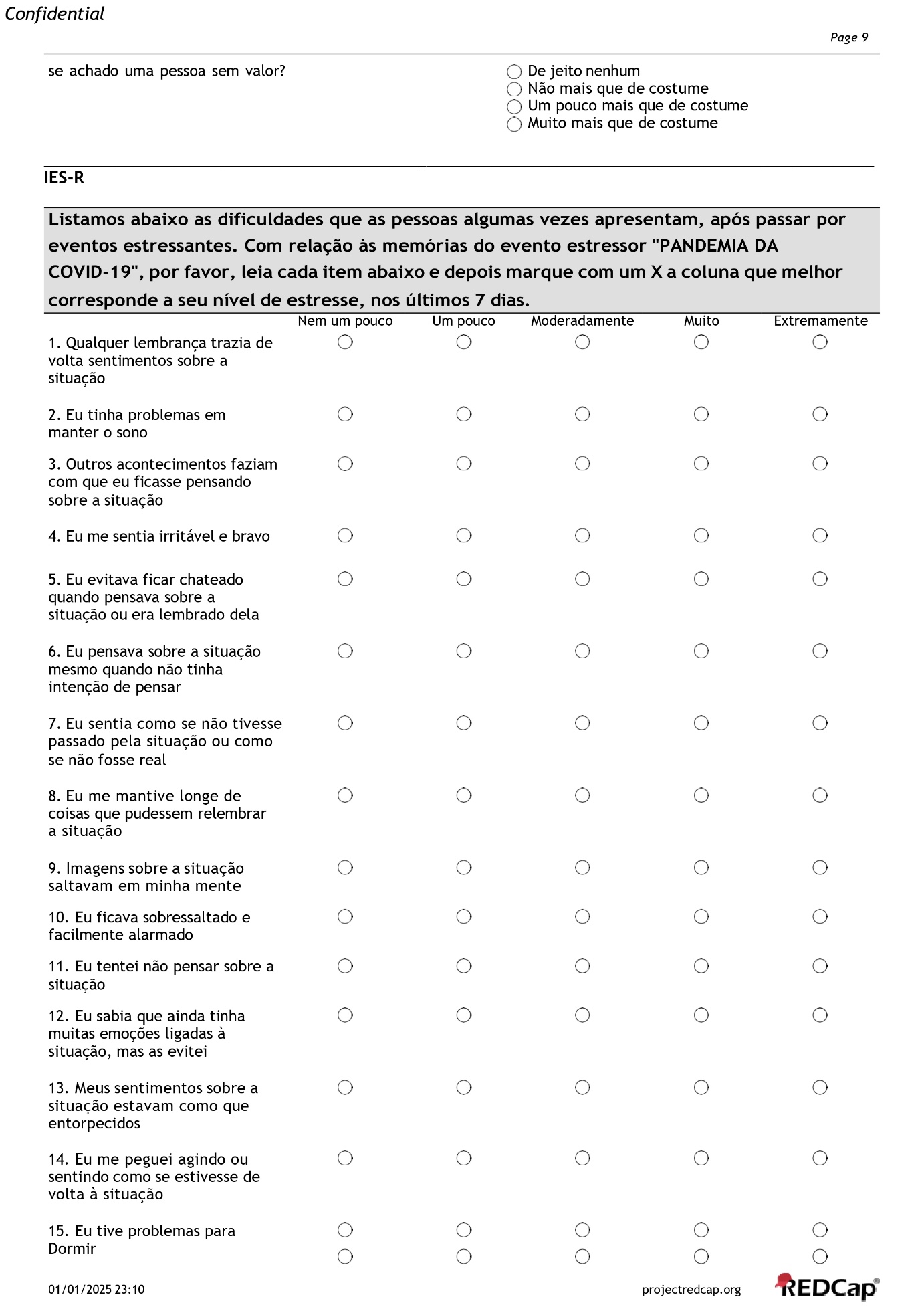

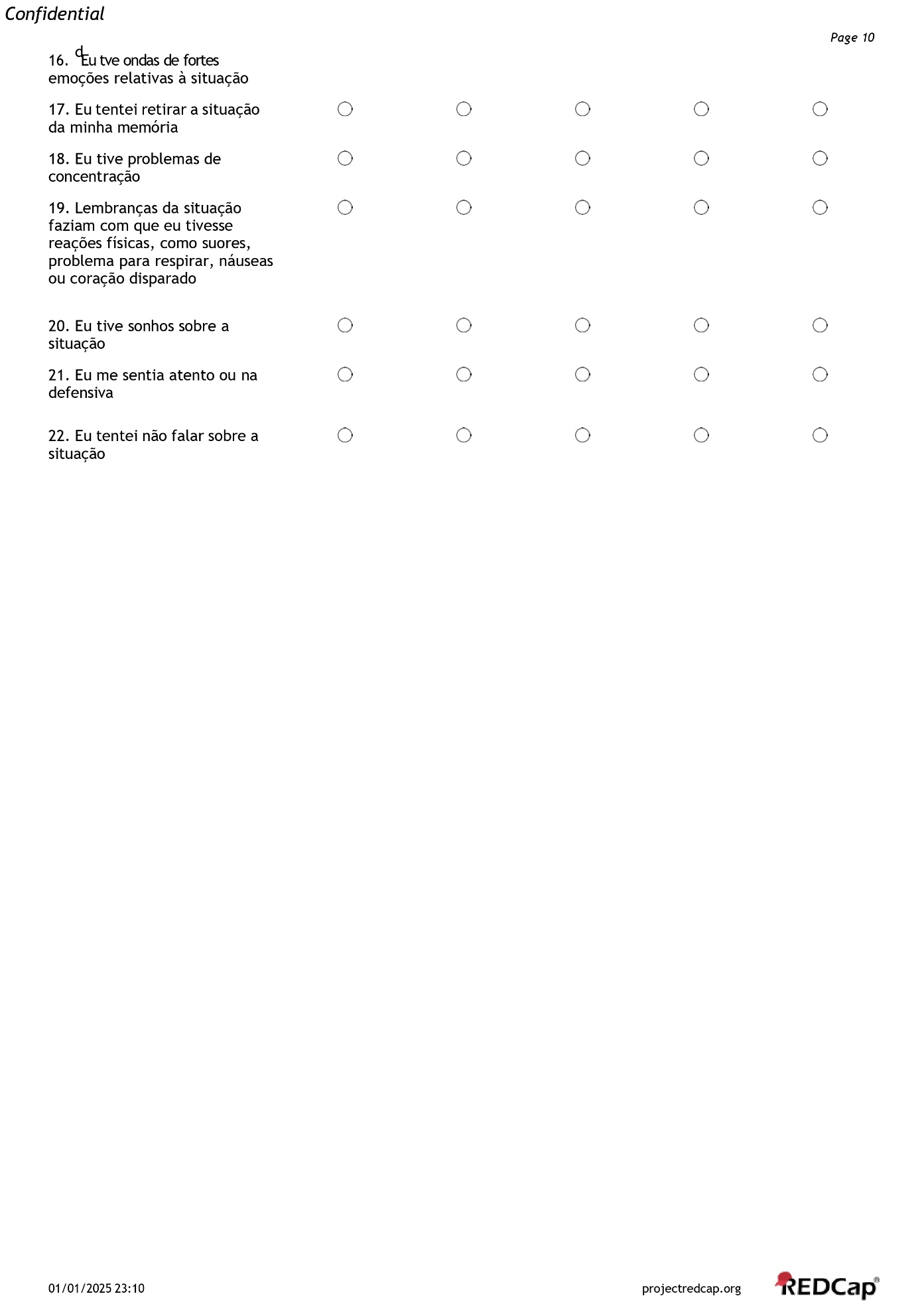

Supplement: Supplementary file 1 [file ijerph-22-00271-s001.zip › ijerph-3388247-supplementary.docx]
